# Genetics of Isolated Hypogonadotropic Hypogonadism: Role of GnRH Receptor and Other Genes

**DOI:** 10.1155/2012/147893

**Published:** 2011-12-21

**Authors:** Karges Beate, Neulen Joseph, de Roux Nicolas, Karges Wolfram

**Affiliations:** ^1^Division of Endocrinology and Diabetes, University Hospital Aachen, RWTH Aachen University, 52074 Aachen, Germany; ^2^Department of Gynecological Endocrinology and Reproductive Medicine, University Hospital Aachen, RWTH Aachen University, 52074 Aachen, Germany; ^3^INSERM U676, Paris Diderot University, Robert Debré Hospital, 75019 Paris, France

## Abstract

Hypothalamic gonadotropin releasing hormone (GnRH) is a key player in normal puberty and sexual development and function. Genetic causes of isolated hypogonadotropic hypogonadism (IHH) have been identified during the recent years affecting the synthesis, secretion, or action of GnRH. Developmental defects of GnRH neurons and the olfactory bulb are associated with hyposmia, rarely associated with the clinical phenotypes of synkinesia, cleft palate, ear anomalies, or choanal atresia, and may be due to mutations of KAL1, FGFR1/FGF8, PROKR2/PROK2, or CHD7. Impaired GnRH secretion in normosmic patients with IHH may be caused by deficient hypothalamic GPR54/KISS1, TACR3/TAC3, and leptinR/leptin signalling or mutations within the GNRH1 gene itself. Normosmic IHH is predominantly caused by inactivating mutations in the pituitary GnRH receptor inducing GnRH resistance, while mutations of the **β**-subunits of LH or FSH are very rare. Inheritance of GnRH deficiency may be oligogenic, explaining variable phenotypes. Future research should identify additional genes involved in the complex network of normal and disturbed puberty and reproduction.

## 1. Introduction

Normal pubertal development and reproductive function depends on the intact release and action of hypothalamic gonadotropin releasing hormone (GnRH). As a precondition, distinct developmental and functional procedures involving the coordinated action of other hypothalamic hormone-receptor systems are required for GnRH disposal. The detailed diagnostic workup of patients with absent or incomplete pubertal development due to gonadotropin deficiency has recently led to the identification of new genetic causes of isolated hypogonadotropic hypogonadism (IHH) [1–7]. These findings currently improve our understanding of how the onset and course of puberty and reproduction are controlled. The precise classification of the underlying defect in the patient with IHH may, in turn, improve the clinical management including choice and timing of therapeutic intervention.

## 2. Normal Onset of Puberty

The hypothalamic GnRH pulse generator constitutes the basis of the CNS control of puberty. GnRH secretion is suppressed during childhood via inhibitory neurotransmitters, mainly gamma aminobutyric acid (GABA) and opioid peptides [8]. After a rest period from approximately two until 8 to 9 years of age, declining inhibitory components and amplifying excitatory transmitters including glutamate and kisspeptin enhance GnRH secretion. 

 The pubertal increase in GnRH secretion is initiated and prompted by changes in transsynaptic and glial inputs to the GnRH neuronal network [8]. Kisspeptins coordinate environmental and metabolic factors for regulation of the hypothalamic-pituitary-gonadal axis through modulation of GnRH, LH, and FSH secretion and steroid feedback [9]. The pulsatile GnRH release from GnRH-containing neurons with frequency and amplitude modulation is the main determinant of system activation with progression into and through puberty.

The stimulatory decapeptide GnRH binds in a hairpin structure to its transmembrane receptor expressed in pituitary gonadotrope cells [10]. The amino- and carboxy-terminal domains of GnRH contribute to receptor binding and activation via extracellular and transmembrane domains inducing conformational changes and signal transduction, thereby inducing synthesis and secretion of luteinizing hormone (LH) and follicle-stimulating hormone (FSH). These gonadotropins bind to their specific receptors in gonads and stimulate synthesis of estrogens and testosterone resulting in clinical signs of puberty. The functional integrity of this hypothalamic-pituitary-gonadal system is the precondition for normal reproductive function [9].

## 3. Causes of Isolated Normosmic Hypogonadotropic Hypogonadism and Kallmann's Syndrome

**Figure 1:**
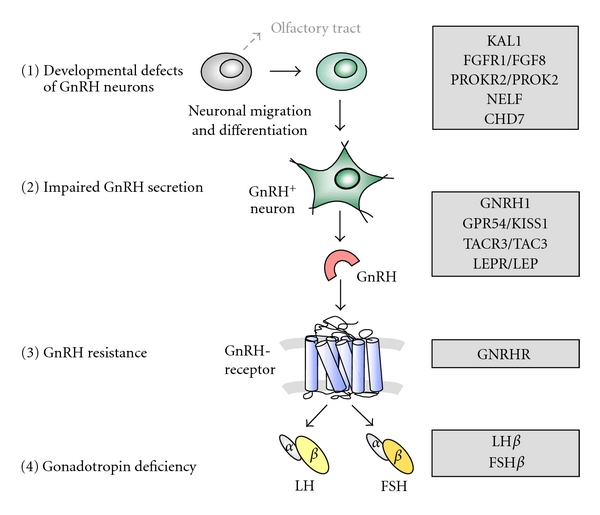
Genetic control of pubertal development. Different levels of GnRH and gonadotropin deficiency due to genetic disorders. (1) Developmental defects of GnRH neurons due to disturbed neuronal migration and differentiation cause aplasia of GnRH neurons and olfactory tract. (2) Impaired GnRH synthesis or secretion is found in the context of functional disorders within the hypothalamus or the GnRH neuron itself. (3) GnRH resistance is caused by inactive GnRH receptor variants localised within the anterior pituitary gland. (4) Gonadotropin deficiency may be due to defect synthesis of LH or FSH *β*-subunits.

IHH is characterised by impaired gonadotropin release in the context of otherwise normal anatomical and functional anterior pituitary function. Serum concentrations for LH, FSH, and sex steroids are inappropriately low in the patient with hypogonadism. Clinical signs and symptoms of hypogonadism include bilateral cryptorchidism in males, absent or incomplete puberty with amenorrhea in females, and infertility. The underlying cause may be due to developmental defects of GnRH neurons, impaired functional activity within GnRH neurons, disturbed interaction between the GnRH ligand and its receptor, or the release of intact gonadotropins (Figure 1).

While aplasia of GnRH neurons occurs in the context of developmental defects of the olfactory bulb, the clinical symptom of anosmia indicates this kind of GnRH deficiency [4, 5, 11–15]. Kallmann's syndrome accounts for 50–52% of cases with IHH, while normosmic IHH is found in 48–50% of cases [16, 17]. Disorders of GnRH release have recently been identified as rare causes of GnRH deficiency in patients with normosmic IHH [2, 3, 7], while inactivating mutations of the GnRH receptor are the most frequent cause for normosmic IHH, especially in familial cases [12, 17–21]. 

## 4. Developmental Abnormalities of GnRH Neurons and Anosmia

**Figure 2:**
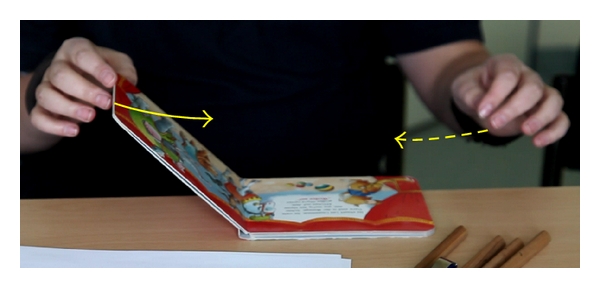
Kallmann's Syndrome. Synkinesia in a patient with KAL1 mutation (c.120_121insC; 122_127del, p.Ala41Glyfs43). When closing a book with one hand (arrow), typical involuntary mirror movement (synkinesia) of the other hand is observed (dotted arrow). This 15-year-old boy presented because of absent puberty. There was orchidopexy at the age of 2 years, disturbed spatial orientation, retarded fine-motor developmental milestones, and anosmia. Tanner P1, G1. LH <0.1 U/L, FSH 0.4 U/L, testosterone 0.2 ng/mL. During GnRH stimulation test, LH 0.5 U/L, FSH 2.1 U/L after 60 minutes. Cranial MRI revealed agenesis of olfactory bulbs and a normal-sized pituitary gland.

Developmental defects of the olfactory bulb and GnRH secreting neurons in patients with Kallmann's syndrome are caused by genetic alterations regulating the migration of GnRH neurons from the forebrain to the hypothalamus (Table 1). The KAL1 encoded protein, anosmin-1, is an adhesion protein involved in synaptogenesis, cell adhesion, and olfactory axonal attraction and olfactory bulb morphogenesis [22]. Deletions and mutations of *KAL1 *account for approximately 10% of Kallmann's syndrome patients [16, 17, 23]. Individuals with KAL1 mutations may present with additional symptoms such as bimanual synkinesia characterised by involuntary “mirror movements” (Figure 2) and renal agenesis [16]. Since *KAL1* is a X-linked gene, familial Kallmann's syndrome occurring only in males suggests a KAL1 defect.

The fibroblast growth factor receptor (FGFR1) gene encodes a tyrosine kinase receptor involved in olfactory bulb development and GnRH neurite outgrowth via FGF signalling and the interaction between FGFR1 and anosmin-1 [22, 24]. Inactivating mutations of this receptor and one of its ligands, fibroblast growth factor 8 (FGF8), have been described in patients with variable degree of hypogonadism mainly with and in few cases without anosmia [4, 14, 15, 22, 25, 26]. In very few subjects with FGFR1 mutations, a complete reversal of GnRH deficiency has been reported [27–29]. Additional clinical signs observed in these individuals include cleft palate or lip, ear anomalies, and tooth agenesis [4, 15, 25, 29]. Heterozygous mutations and deletions of the FGFR1/FGF8 system account for approximately 10% of Kallmann's syndrome and normosmic idiopathic hypogonadotropic hypogonadism [14, 25, 26].

The prokineticin receptor 2 (PROKR2), a heptahelical transmembrane G protein-coupled receptor, and its ligand prokineticin 2 (PROK2) are expressed within the CNS including olfactory system, arcuate nucleus, suprachiasmatic nuclei, and median eminence [30, 31]. The PROKR2/PROK2 system is involved in olfactory bulb development and in GnRH neuron migration [32]. Heterozygous, compound heterozygous and homozygous inactivating mutations have been described within the PROKR2/PROK2 system, accounting for less than 10% of individuals with Kallmann's syndrome and normosmic GnRH deficiency [5, 32–34]. One patient with a heterozygous PROKR2 mutation has been reported with reversal of hypogonadism after treatment with testosterone [28, 34].

The human nasal embryonic LHRH factor (NELF) gene is a candidate gene for Kallmann's syndrome because of its association with axonal guidance of olfactory and GnRH neurons in mice [35]. Heterozygous mutations within the NELF gene have been reported in few patients with Kallmann's syndrome [36–38]. So far, the role of NELF in human reproduction is unclear, but NELF may be a critical modifier gene that orchestrates GnRH deficiency in conjunction with other pathogenic genes [36].

**Figure 3:**
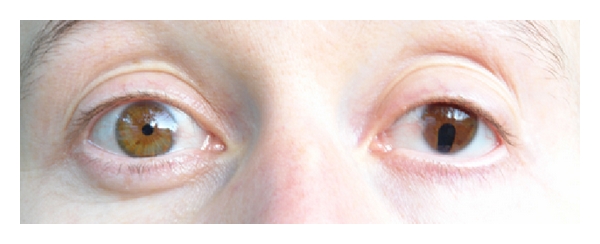
Iris coloboma as a typical characteristic of CHARGE syndrome. A woman with hypogonadotropic hypogonadism and CHARGE syndrome (CHD7 mutation c.4787A>G, p.Asp1596Gly) initially presented at the age of 16 years because of absent puberty. There was a history of choanal atresia, deafness, learning disorders, and anosmia. Tanner B1, P2. LH 0.2 U/L, FSH 0.54 U/L, estradiol 24 pg/mL. During GnRH stimulation test, LH 1.61 U/L, FSH 1.86 U/L.

Mutations within the chromodomain helicase DNA-binding protein 7 (CHD7) have been identified in patients with CHARGE association, a syndrome in which hypogonadotropic hypogonadism and hyposmia are associated with choanal atresia, coloboma of the iris (Figure 3), cardiovascular malformations, retardation of mental and somatic development, and ear anomalies [13, 39]. Recently, CHD7 heterozygous mutations have been identified in subjects with hypogonadotropic hypogonadism, with and without anosmia [40]. Patients presenting some of the CHARGE syndrome features are more likely to carry CHD7 mutations [41]. CHD7 mutations are found in 5 to 10% of subjects initially classified as Kallmann's syndrome and normosmic IHH patients [40, 41].

Very recently, heterozygous mutations of *WDR11*, encoding a WD protein interacting with the transcription factor EMX1, have been identified in six patients with Kallmann's syndrome or idiopathic hypogonadotropic hypogonadism  [42]. The interaction between WDR11 and EMX1 is critical for the development of olfactory neurons while WDR11 missense alterations reduce or abolish this interaction [42]. It was concluded from these results that disturbed pubertal development in these patients is caused by deficient WDR11 protein interaction [42].

## 5. Defects of GnRH Release and Synthesis

The identification of inactivating mutations within the G-protein-coupled receptor 54 (GPCR54/KISSR) gene has demonstrated the role of kisspeptin, the ligand of GPR54, in the control of GnRH secretion [2, 3, 43–45] (Table 1). GPR54/KISSR is a heptahelical transmembrane receptor, expressed at the surface of GnRH neurons. GPR54 activation via kisspeptin induces GnRH secretion [9]. Neuroendocrine profiles of subjects with GPR54/KISSR mutations revealed low amplitude of LH pulses, suggesting low degree of endogeneous GnRH secretion [3, 46]. Male patients may present at birth with micropenis and cryptorchidism and undetectable gonadotropin levels [43, 46]. GPR54/KISSR mutations account for 2–5% of normosmic IHH [2, 3, 43]. Until now, mutations within the gene of the ligand of GPR54/KISSR, KISS1, have not been described in patients with IHH.

 Very recently, homozygous loss-of-function mutations in TAC3, encoding neurokinin B and its heptahelical transmembrane G-protein-coupled receptor TACR3, have been detected in patients with normosmic IHH [7, 47, 48]. Affected subjects showed very low basal LH secretion with nonpulsatile pattern while pulsatile GnRH treatment normalised LH release and circulating sex steroids [47]. These findings indicate a crucial role of NKB, via its receptor NK3R, in hypothalamic GnRH release [47]. The majority of male patients with TACR3/TAC3 mutations presented with micropenis and lack or pubertal development while recovery of GnRH deficiency was observed in a significant number of male and female adult patients [48]. These observations support the importance of the TACR3/TAC3 signaling during the neonatal period and puberty while its role seems less critical in adulthood [48].

The role of leptin for pubertal development and reproduction has been demonstrated in leptin-null (ob/ob) mice in which leptin administration accelerates puberty and normalises reproductive dysfunction [49]. Leptin, encoded by LEP, is a fat-derived hormone regulating food intake, energy expenditure, and hypothalamic reproductive function. Inactivating mutations in LEP or its receptor LEPR, a single transmembrane-domain receptor of the cytokine receptor family, have been described in patients with hypogonadism and obesity [50–52]. These loss-of-function mutations are rare causes of normosmic IHH. Treatment with recombinant leptin reconstitutes gonadotropin secretion and menstrual cycles in females with amenorrhea due to congenital leptin deficiency [53] or hypothalamic amenorrhea [54].

The most obvious candidate gene for patients with hypogonadotropic hypogonadism was GnRH itself after description of the hypogonadal mouse model with homozygous deletion within the GNRH1 gene [55, 56]. However, several studies initially failed to identify GNRH1 gene mutations in humans with hypogonadotropic hypogonadism [57, 58]. Very recently, homozygous frameshift mutations within the GNRH1 gene, encoding the preprohormone of GnRH, have been identified in patients with IHH [6, 59]. In accordance with the critical role of GnRH, male patients presented with severe hypogonadism including micropenis. *GNRH1* mutations are rare causes of normosmic isolated GnRH deficiency.

## 6. GnRH Resistance and Gonadotropin Deficiency

Binding of GnRH to its heptahelical transmembrane receptor in the pituitary gland induces receptor activation and signal transduction, finally resulting in secretion of gonadotropins. Since the first description of loss-of-function mutation in the GnRH receptor (GnRHR) [1], many inactivating mutations have been found within the extracellular, transmembrane and intracellular domains of the receptor [11, 19–21] leading to impaired GnRH action (Figure 1). Depending on the degree of functional impairment, these patients present with complete absence of pubertal development or with incomplete puberty [19]. Loss-of-function mutations within the GnRH receptor are the most frequent cause of autosomal-recessive IHH, accounting for 16% to 40% of patients [18, 21, 60]. Since these patients are resistant to GnRH, the effective fertility treatment is achieved with gonadotropins.

Mutations of the *β*-subunits of luteinizing hormone (LH) or follicle-stimulating hormone (FSH) are rare causes of hypogonadotropic hypogonadism. LH and FSH are glycoprotein hormones, as thyroid-stimulating hormone and human chorionic gonadotropin (hCG). These heterodimeric hormones consist of a common *α*-subunit and a specific *β*-subunit, encoded by separate genes. Females with inactivating mutations of the LH *β*-subunit present with normal puberty, with normal or late menarche followed by oligo- or amenorrhea and infertility due to lack of ovulation [61]. Ovaries in affected women may be enlarged with cysts [62]. Males with inactivating mutations of the LH *β*-subunit have absent pubertal development due to testosterone deficiency and azoospermia in adulthood because of Leydig-cell hypoplasia [61–63]. Testosterone replacement may result in an increase of testicular volume in the context of high FSH levels [61]. Individuals with inactivating FSH*β* mutations present with incomplete pubertal development and primary amenorrhea in females and azoospermia in males [64–66]. Treatment with recombinant FSH induces ovulation but was associated with signs of ovarian hyperstimulation which may be explained by high pretreatment LH levels [67].

## 7. Clinical Implications

Since pulsatile GnRH secretion is required for descent of the testis in the male fetus, patients with gonadotropin deficiency during fetal life may present with cryptorchidism and variable degree of male undervirilisation. Additional symptoms such as impaired sense of smell, bilateral synkinesia, cleft palate, or choanal atresia are suspicious for specific congenital diseases associated with GnRH deficiency (Table 1). Absent or incomplete pubertal development leading to detailed diagnostic workup may identify congenital GnRH or gonadotropin deficiency.

Hormonal replacement therapy during adolescence is frequently delayed, although earlier signs and symptoms of the patient would have predicted hypogonadotropic hypogonadism. Since hormonal induction of puberty does not always require the definite identification of the underlying cause of GnRH or gonadotropin deficiency, some individuals are investigated only later in life because of infertility. In most cases of IHH, gonadotropin treatment induces ovulation and spermatogenesis [68, 69], while patients with inactive GnRHR variants will not respond to normal doses of GnRH treatment [70, 71]. This GnRH resistance has been overcome with higher GnRH doses in one subject with partially inactivated GnRH receptor mutations [72].

In addition to absent or incomplete pubertal development and infertility, further clinical variants of GnRH and gonadotropin deficiency associated with genetic variants have been recently observed. These variants include adult-onset idiopathic hypogonadotropic hypogonadism [73], functional hypothalamic amenorrhea [74], and spontaneous reversals of well-established GnRH deficiency following long-term therapy with testosterone [28]. Although the mechanisms of reversal of hypogonadotropic hypogonadism are unclear, it is speculated that GnRH neuron plasticity in adults may be modulated by sex steroids [28]. Brief discontinuation of hormonal replacement may, therefore, be reasonable to assess if hypogonadotropic hypogonadism is reversible or persistent [28].

After a detailed individual and family history and physical examination evaluating the degree of hypogonadism and presence of associated clinical symptoms (e.g., Figures 2 and 3), a molecular genetic analysis enables in many cases definition of the underlying defect. Monogenic, digenic, or even oligogenic inheritance of GnRH deficiency has been observed explaining the variable phenotypic spectrum [17, 28, 36]. Alterations in two or more distinct genes in one patient may induce a more severe phenotype than a single-gene mutation and lead to the overlap of two or more clinical syndromes. Rare genetic variants may further contribute to the susceptibility of individuals to functional changes in GnRH secretion such as hypothalamic amenorrhea, a common multifactorial disease [74]. Genetic counselling is offered in case of genetic diagnosis to first-degree family members. However, approximately 60–70% of cases with Kallmann's syndrome and 50% of patients with normosmic IHH are of unknown origin [60]. These patients and families should be encouraged to participate in ongoing research projects including DNA biobanking. In any case, early diagnosis of GnRH deficiency during childhood represents the requisite for induction of puberty in due time.

## 8. Hormonal Treatment of IHH

The hormonal induction of puberty in a hypogonadal adolescent aims to mimic normal pubertal development. Hormone replacement in adolescents is usually initiated with low dose of sex steroids and augmented over 3 to 5 years until mature status is reached. In girls, estradiol orally is preferred, starting with one-sixths of the adult dose daily, increasing every 6 months by 1/6 and adding gestagens from the second year on day 1 to 12 of each month [75–77]. In boys, testosterone replacement is initiated most frequently with testosterone enanthate 50 mg per month intramuscularly, with increasing dose every 6 months until 250 mg is given every 3 weeks in the third year. While testosterone treatment effectively induces virilisation including penile growth, pubic and male hair and beard growth, change of voice, libido, and pubertal growth spurt, testicular volume remains small, lacking spermatogenesis. LH stimulates intratesticular testosterone secretion by Leydig cells inhibiting Anti-Müller's hormone production of the Sertoli cells, FSH induces testis growth via proliferation of seminiferous tubules, and both stimulate Inhibin B secretion by the Sertoli cells and sperm maturation. Therefore, induction of puberty using gonadotropins or pulsatile GnRH seems a more physiologic approach in the adolescent with hypogonadotropic hypogonadism and has been successfully used [78–82]. To further assess the benefit of GnRH or gonadotropin treatment for pubertal induction, prospective randomised trials are needed.

During adulthood testosterone replacement may be continued by daily transdermal application of testosterone gel or injection of the long-acting testosterone undecanoate intramuscularly every 3 months. Fertility treatment usually requires gonadotropin treatment with hCG and FSH or may alternatively, initiated by pulsatile GnRH treatment [80–82]. GnRH given every 90 minutes by a subcutaneous placed pump is the most physiologic therapy of GnRH deficiency, except in case of GnRH resistance, but is associated with higher costs and technical support. In rare cases of leptin deficiency, specific leptin treatment has been effective for treatment of hypogonadism [53, 54]. In general, long-term replacement of sex steroids is required not only for sexual and reproductive function but also for bone health and metabolic (glucose and fat) integrity in patients with hypogonadotropic hypogonadism.

## 9. Conclusion

The discovery of new genetic causes of hypogonadotropic hypogonadism gave new insights into the regulation of puberty and reproduction in humans. With the identification of genetic variants in GnRH-deficient patients, it became clear that monogenic, digenic, and oligogenic traits of inheritance may explain the variable phenotypic spectrum. In more than 50% of patients with IHH, the underlying defect is still unknown, demonstrating the need for further research activity in this field. The precise diagnosis facilitates appropriate treatment and counselling in affected patients. Established treatment procedures for hormonal induction of puberty might be reconsidered, since pulsatile GnRH and gonadotropin treatment are effective and more physiologic alternatives. To investigate the benefit of different therapeutic options on quality of life and fertility, prospective randomised controlled trials with long-term followup have to be conducted. For these future research directions, national and international scientific networking will be advantageous.

## Figures and Tables

**Table 1:** Genetic causes of Kallmann's syndrome (KS) and normosmic isolated hypogonadotropic hypogonadism (IHH).

Gene	Gene product	Function	Inheritance	Clinical phenotype	Associated clinical phenotype
KAL1	Anosmin-1	Cell adhesion	X-linked	KS	Anosmia, bimanual synkinesis, renal agenesis
FGFR1	Fibroblast-growth-factor receptor 1	Tyrosine kinase receptor	AD	KS or IHH	Anosmia, cleft lip or palate, ear anomalies, tooth agenesis
FGF8	Fibroblast growth factor 8	Ligand of FGFR1	AD	KS or IHH	
NELF	Nasal embryonic LHRH factor	Neuronal migration	AD	KS	Anosmia
CHD7	Chromodomain- helicase-DNA-binding protein 7	DNA-binding protein, neural crest development	AD	KS or IHH	CHARGE syndrome: anosmia, coloboma, heart anomaly, choanal atresia, retardation, ear abnormalities
PROKR2	Prokineticin receptor 2	GPCR	AD AR	KS or IHH	Anosmia
PROK2	Prokineticin 2	Ligand of PROKR2	AD AR	KS or IHH	Anosmia
WDR11	WD protein	Interaction with EMX1	AD	KS or IHH	Anosmia
GPR54/KISS1R	Kisspeptin-1 receptor	GPCR	AR	IHH	None
TACR3	Neurokinin B receptor	GPCR	AR	IHH	None
TAC3	Neurokinin B	Ligand of TACR3	AR	IHH	None
LEPR	leptin receptor	Single transmembrane-domain receptor	AR	IHH	Obesity
LEP	leptin	Fat-regulating hormone	AR	IHH	Obesity
GNRH1	GnRH	Release of LH and FSH	AR	IHH	None
GNRHR	GnRH receptor	GPCR	AR	IHH	None
LH*β*	*β*-subunit of LH	Ligand of LH/CG receptor	AR	IHH	None
FSH*β*	*β*-subunit of FSH	Ligand of FSH receptor	AR	IHH	None

GPCR: heptahelical transmembrane G-protein-coupled receptor, AD: autosomal dominant.

AR: autosomal recessive.

## References

[1] De Roux N, Young J, Misrahi M (1997). A family with hypogonadotropic hypogonadism and mutations in the gonadotropin-releasing hormone receptor. *New England Journal of Medicine*.

[2] De Roux N, Genin E, Carel JC, Matsuda F, Chaussain JL, Milgrom E (2003). Hypogonadotropic hypogonadism due to loss of function of the KiSS1-derived peptide receptor GPR54. *Proceedings of the National Academy of Sciences of the United States of America*.

[3] Seminara SB, Messager S, Chatzidaki EE (2003). The GPR54 gene as a regulator of puberty. *New England Journal of Medicine*.

[4] Dodé C, Levilliers J, Dupont JM (2003). Loss-of-function mutations in FGFR1 cause autosomal dominant Kallmann syndrome. *Nature Genetics*.

[5] Dodé C, Teixeira L, Levilliers J (2006). Kallmann syndrome: mutations in the genes encoding prokineticin-2 and prokineticin receptor-2.. *PLoS genetics*.

[6] Chan YM, De Guillebon A, Lang-Muritano M (2009). GNRH1 mutations in patients with idiopathic hypogonadotropic hypogonadism. *Proceedings of the National Academy of Sciences of the United States of America*.

[7] Topaloglu AK, Reimann F, Guclu M (2009). TAC3 and TACR3 mutations in familial hypogonadotropic hypogonadism reveal a key role for Neurokinin B in the central control of reproduction. *Nature Genetics*.

[8] Ojeda SR, Lomniczi A, Mastronardi C (2006). Minireview: the neuroendocrine regulation of puberty: Is the time ripe for a systems biology approach?. *Endocrinology*.

[9] Roseweir AK, Millar RP (2009). The role of kisspeptin in the control of gonadotrophin secretion. *Human Reproduction Update*.

[10] Millar RP, Lu Z-L, Pawson AJ, Flanagan CA, Morgan K, Maudsley SR (2004). Gonadotropin-releasing hormone receptors. *Endocrine Reviews*.

[11] Karges B, de Roux N (2005). Molecular genetics of isolated hypogonadotropic hypogonadism and Kallmann syndrome. *Endocrine development*.

[12] Crosignani PG, Collins J, Diedrich K (2008). Genetic aspects of female reproduction. *Human Reproduction Update*.

[13] Pinto G, Abadie V, Mesnage R (2005). CHARGE syndrome includes hypogonadotropic hypogonadism and abnormal olfactory bulb development. *Journal of Clinical Endocrinology and Metabolism*.

[14] Pitteloud N, Meysing A, Quinton R (2006). Mutations in fibroblast growth factor receptor 1 cause Kallmann syndrome with a wide spectrum of reproductive phenotypes. *Molecular and Cellular Endocrinology*.

[15] Dodé C, Fouveaut C, Mortier G (2007). Novel FGFR1 sequence variants in Kallmann syndrome, and genetic evidence that the FGFR1c isoform is required in olfactory bulb and palate morphogenesis. *Human mutation*.

[16] Quinton R, Duke VM, Robertson A (2001). Idiopathic gonadotrophin deficiency: genetic questions addressed through phenotypic characterization. *Clinical Endocrinology*.

[17] Sykiotis GP, Plummer L, Hughes VA (2010). Oligogenic basis of isolated gonadotropin-releasing hormone deficiency. *Proceedings of the National Academy of Sciences of the United States of America*.

[18] Beranova M, Oliveira LMB, Bédécarrats GY (2001). Prevalence, phenotypic spectrum, and modes of inheritance of gonadotropin-releasing hormone receptor mutations in idiopathic hypogonadotropic hypogonadism. *Journal of Clinical Endocrinology and Metabolism*.

[19] Karges B, Karges W, de Roux N (2003). Clinical and molecular genetics of the human GnRH receptor. *Human Reproduction Update*.

[20] Karges B, Karges W, Mine M (2003). Mutation Ala171Thr stabilizes the gonadotropin-releasing hormone receptor in its inactive conformation, causing familial hypogonadotropic hypogonadism. *Journal of Clinical Endocrinology and Metabolism*.

[21] Chevrier L, Guimiot F, de Roux N (2011). GnRH receptor mutations in isolated gonadotropic deficiency: an update in 2011. *Molecular and Cellular Endocrinology*.

[22] Kim SH, Hu Y, Cadman S, Bouloux P (2008). Diversity in fibroblast growth factor receptor 1 regulation: learning from the investigation of Kallmann syndrome. *Journal of Neuroendocrinology*.

[23] Oliveira LMB, Seminara SB, Beranova M (2001). The importance of autosomal genes in Kallmann syndrome: genotype-phenotype correlations and neuroendocrine characteristics. *Journal of Clinical Endocrinology and Metabolism*.

[24] Beenken A, Mohammadi M (2009). The FGF family: biology, pathophysiology and therapy. *Nature Reviews Drug Discovery*.

[25] Pitteloud N, Acierno JS, Meysing A (2006). Mutations in fibroblast growth factor receptor 1 cause both Kallmann syndrome and normosmic idiopathic hypogonadotropic hypogonadism. *Proceedings of the National Academy of Sciences of the United States of America*.

[26] Trarbach EB, Costa EMF, Versiani B (2006). Novel fibroblast growth factor receptor 1 mutations in patients with congenital hypogonadotropic hypogonadism with and without anosmia. *Journal of Clinical Endocrinology and Metabolism*.

[27] Pitteloud N, Acierno JS, Meysing AU, Dwyer AA, Hayes FJ, Crowley WF (2005). Reversible kallmann syndrome, delayed puberty, and isolated anosmia occurring in a single family with a mutation in the fibroblast growth factor receptor 1 gene. *Journal of Clinical Endocrinology and Metabolism*.

[28] Raivio T, Falardeau J, Dwyer A (2007). Reversal of idiopathic hypogonadotropic hypogonadism. *New England Journal of Medicine*.

[29] Falardeau J, Chung WCJ, Beenken A (2008). Decreased FGF8 signaling causes deficiency of gonadotropin-releasing hormone in humans and mice. *Journal of Clinical Investigation*.

[30] Ng KL, Li JD, Cheng MY, Leslie FM, Lee AC, Zhou QY (2005). Neuroscience: dependence of olfactory bulb neurogenesis on prokineticin 2 signaling. *Science*.

[31] Matsumoto SI, Yamazaki C, Masumoto KH (2006). Abnormal development of the olfactory bulb and reproductive system in mice lacking prokineticin receptor PKR2. *Proceedings of the National Academy of Sciences of the United States of America*.

[32] Martin C, Balasubramanian R, Dwyer AA (2011). The role of the prokineticin 2 pathway in human reproduction: evidence from the study of human and murine gene mutations. *Endocrine Reviews*.

[33] Pitteloud N, Zhang C, Pignatelli D (2007). Loss-of-function mutation in the prokineticin 2 gene causes Kallmann syndrome and normosmic idiopathic hypogonadotropic hypogonadism. *Proceedings of the National Academy of Sciences of the United States of America*.

[34] Cole LW, Sidis Y, Zhang C (2008). Mutations in prokineticin 2 and prokineticin receptor 2 genes in human gonadotrophin-releasing hormone deficiency: molecular genetics and clinical spectrum. *Journal of Clinical Endocrinology and Metabolism*.

[35] Kramer PR, Wray S (2000). Novel gene expressed in nasal region influences outgrowth of olfactory axons and migration of luteinizing hormone-releasing hormone (LHRH) neurons. *Genes and Development*.

[36] Pitteloud N, Quinton R, Pearce S (2007). Digenic mutations account for variable phenotypes in idiopathic hypogonadotropic hypogonadism. *Journal of Clinical Investigation*.

[37] Miura K, Acierno JS, Seminara SB (2004). Characterization of the human nasal embryonic LHRH factor gene, NELF, and a mutation screening among 65 patients with idiopathic hypogonadotropic hypogonadism (IHH). *Journal of Human Genetics*.

[38] Xu N, Kim H-G, Bhagavath B (2011). Nasal embryonic LHRH factor (NELF) mutations in patients with normosmic hypogonadotropic hypogonadism and Kallmann syndrome. *Fertility and Sterility*.

[39] Vissers LELM, Van Ravenswaaij CMA, Admiraal R (2004). Mutations in a new member of the chromodomain gene family cause CHARGE syndrome. *Nature Genetics*.

[40] Kim HG, Kurth I, Lan F (2008). Mutations in CHD7, encoding a chromatin-remodeling protein, cause idiopathic hypogonadotropic hypogonadism and Kallmann syndrome. *American Journal of Human Genetics*.

[41] Jongmans MCJ, van Ravenswaaij-Arts CMA, Pitteloud N (2009). CHD7 mutations in patients initially diagnosed with Kallmann syndrome—the clinical overlap with CHARGE syndrome. *Clinical Genetics*.

[42] Kim HG, Ahn JW, Kurth I (2010). WDR11, a WD protein that interacts with transcription factor EMX1, is mutated in idiopathic hypogonadotropic hypogonadism and Kallmann syndrome. *American Journal of Human Genetics*.

[43] Semple RK, Achermann JC, Ellery J (2005). Two novel missense mutations in G protein-coupled receptor 54 in a patient with hypogonadotropic hypogonadism. *Journal of Clinical Endocrinology and Metabolism*.

[44] Pallais JC, Bo-Abbas Y, Pitteloud N, Crowley WF, Seminara SB (2006). Neuroendocrine, gonadal, placental, and obstetric phenotypes in patients with IHH and mutations in the G-protein coupled receptor, GPR54. *Molecular and Cellular Endocrinology*.

[45] Nimri R, Lebenthal Y, Lazar L (2011). A novel loss-of-function mutation in GPR54/KISS1R leads to hypogonadotropic hypogonadism in a highly consanguineous family. *Journal of Clinical Endocrinology and Metabolism*.

[46] Tenenbaum-Rakover Y, Commenges-Ducos M, Iovane A, Aumas C, Admoni O, De Roux N (2007). Neuroendocrine phenotype analysis in five patients with isolated hypogonadotropic hypogonadism due to a L102P inactivating mutation of GPR54. *Journal of Clinical Endocrinology and Metabolism*.

[47] Young J, Bouligand J, Francou B (2010). TAC3 and TACR3 defects cause hypothalamic congenital hypogonadotropic hypogonadism in humans. *Journal of Clinical Endocrinology and Metabolism*.

[48] Gianetti E, Tusset C, Noel SD (2010). TAC3/TACR3 mutations reveal preferential activation of gonadotropin- releasing hormone release by neurokinin B in neonatal life followed by reversal in adulthood. *Journal of Clinical Endocrinology and Metabolism*.

[49] Chehab FF, Lim ME, Lu R (1996). Correction of the sterility defect in homozygous obese female mice by treatment with the human recombinant leptin. *Nature Genetics*.

[50] Clément K, Vaisse C, Lahlou N (1998). A mutation in the human leptin receptor gene causes obesity and pituitary dysfunction. *Nature*.

[51] Strobel A, Issad T, Camoin L, Ozata M, Strosberg AD (1998). A leptin missense mutation associated with hypogonadism and morbid obesity. *Nature genetics*.

[52] Fischer-Posovszky P, Von Schnurbein J, Moepps B (2010). A new missense mutation in the leptin gene causes mild obesity and hypogonadism without affecting T cell responsiveness. *Journal of Clinical Endocrinology and Metabolism*.

[53] Licinio J, Caglayan S, Ozata M (2004). Phenotypic effects of leptin replacement on morbid obesity, diabetes mellitus, hypogonadism, and behavior in leptin-deficient adults. *Proceedings of the National Academy of Sciences of the United States of America*.

[54] Welt CK, Chan JL, Bullen J (2004). Recombinant human leptin in women with hypothalamic amenorrhea. *New England Journal of Medicine*.

[55] Mason AJ, Hayflick JS, Zoeller RT (1986). A deletion truncating the gonadotropin-releasing hormone gene is responsible for hypogonadism in the hpg mouse. *Science*.

[56] Mason AJ, Pitts SL, Nikolics K (1986). The hypogonadal mouse: reproductive functions restored by gene therapy. *Science*.

[57] Weiss J, Crowley WF, Jameson JL (1989). Normal structure of the gonadotropin-releasing hormone (GnRH) gene in patients with GnRH deficiency and idiopathic hypogonadotropic hypogonadism. *Journal of Clinical Endocrinology and Metabolism*.

[58] Bo-Abbas Y, Acierno JS, Shagoury JK, Crowley WF, Seminara SB (2003). Autosomal recessive idiopathic hypogonadotropic hypogonadism: genetic analysis excludes mutations in the gonadotropin-releasing hormone (GnRH) and GnRH receptor genes. *Journal of Clinical Endocrinology and Metabolism*.

[59] Bouligand J, Ghervan C, Tello JA (2009). Isolated familial hypogonadotropic hypogonadism and a GNRH1 mutation. *New England Journal of Medicine*.

[60] Bianco SDC, Kaiser UB (2009). The genetic and molecular basis of idiopathic hypogonadotropic hypogonadism. *Nature Reviews Endocrinology*.

[61] Lofrano-Porto A, Barra GB, Giacomini LA (2007). Luteinizing hormone beta mutation and hypogonadism in men and women. *New England Journal of Medicine*.

[62] Themmen APN, Huhtaniemi IT (2000). Mutations of gonadotropins and gonadotropin receptors: elucidating the physiology and pathophysiology of pituitary-gonadal function. *Endocrine Reviews*.

[63] Weiss J, Axelrod L, Whitcomb RW, Harris PE, Crowley WF, Jameson JL (1992). Hypogonadism caused by a single amino acid substitution in the *β* subunit of luteinizing hormone. *Obstetrical and Gynecological Survey*.

[64] Matthews CH, Borgato S, Beck-Peccoz P (1993). Primary amenorrhoea and infertility due to a mutation in the *β*-subunit of follicle-stimulating hormone. *Nature Genetics*.

[65] Layman LC, Lee EJ, Peak DB (1997). Delayed puberty and hypogonadism caused by mutations in the follicle- stimulating hormone *β*-subunit gene. *New England Journal of Medicine*.

[66] Layman LC, Porto ALA, Xie J (2002). FSH*β* gene mutations in a female with partial breast development and a male sibling with normal puberty and azoospermia. *Journal of Clinical Endocrinology and Metabolism*.

[67] Kottler ML, Chou YY, Chabre O (2010). A new FSH*β* mutation in a 29-year-old woman with primary amenorrhea and isolated FSH deficiency: functional characterization and ovarian response to human recombinant FSH. *European Journal of Endocrinology*.

[68] Silveira LFG, MacColl GS, Bouloux PMG (2002). Hypogonadotropic hypogonadism. *Seminars in Reproductive Medicine*.

[69] Zitzmann M, Nieschlag E (2000). Hormone substitution in male hypogonadism. *Molecular and Cellular Endocrinology*.

[70] Pralong FP, Gomez F, Castillo E (1999). Complete hypogonadotropic hypogonadism associated with a novel inactivating mutation of the gonadotropin-releasing hormone receptor. *Journal of Clinical Endocrinology and Metabolism*.

[71] Layman LC, McDonough PG, Cohen DP, Maddox M, Tho SPT, Reindollar RH (2001). Familial gonadotropin-releasing hormone resistance and hypogonadotropic hypogonadism in a family with multiple affected individuals. *Fertility and Sterility*.

[72] Seminara SB, Beranova M, Oliveira LMB, Martin KA, Crowley WF, Hall JE (2000). Successful use of pulsatile gonadotropin-releasing hormone (GnRH) for ovulation induction and pregnancy in a patient with GnRH receptor mutations. *Journal of Clinical Endocrinology and Metabolism*.

[73] Dwyer AA, Hayes FJ, Plummer L, Pitteloud N, Crowley WF (2010). The long-term clinical follow-up and natural history of men with adult-onset idiopathic hypogonadotropic hypogonadism. *Journal of Clinical Endocrinology and Metabolism*.

[74] Caronia LM, Martin C, Welt CK (2011). A genetic basis for functional hypothalamic amenorrhea. *New England Journal of Medicine*.

[75] Ranke MB, Dörr HG (2009). Sex steroid replacement therapy in adolescents with hypogonadism. Expert workshop consensus. *Gynakologische Endokrinologie*.

[76] Drobac S, Rubin K, Rogol AD, Rosenfield RL (2006). A Workshop on pubertal hormone replacement options in the United States. *Journal of Pediatric Endocrinology and Metabolism*.

[77] Kiess W, Conway G, Ritzen M (2002). Induction of puberty in the hypogonadal girl—practices and attitudes of pediatric endocrinologists in Europe. *Hormone Research*.

[78] Raivio T, Toppari J, Perheentupa A, McNeily AS, Dunkel L (1997). Treatment of prepubertal gonadotrophin-deficient boys with recombinant human follicle-stimulating hormone. *Lancet*.

[79] Barrio R, De Luis D, Alonso M, Lamas A, Moreno JC (1999). Induction of puberty with human chorionic gonadotropin and follicle-stimulating hormone in adolescent males with hypogonadotropic hypogonadism. *Fertility and Sterility*.

[80] Büchter D, Behre HM, Kliesch S, Nieschlag E (1998). Pulsatile GnRH or human chorionic gonadotropin/human menopausal gonadotropin as effective treatment for men with hypogonadotropic hypogonadism: a review of 42 cases. *European Journal of Endocrinology*.

[81] Delemarre-Van De Waal HA (2004). Application of gonadotropin releasing hormone in hypogonadotropic hypogonadism—diagnostic and therapeutic aspects. *European Journal of Endocrinology, Supplement*.

[82] Liu PY, Baker HWG, Jayadev V, Zacharin M, Conway AJ, Handelsman DJ (2009). Induction of spermatogenesis and fertility during gonadotropin treatment of Gonadotropin-Deficient infertile men: predictors of fertility outcome. *Journal of Clinical Endocrinology and Metabolism*.

